# Marketing Responses to the Taxation of Soft Drinks: Comment on "Understanding Marketing Responses to a Tax on Sugary Drinks: A Qualitative Interview Study in the United Kingdom, 2019"

**DOI:** 10.34172/ijhpm.2023.7612

**Published:** 2023-06-20

**Authors:** Leigh Sparks

**Affiliations:** Stirling Management School, University of Stirling, Stirling, UK

**Keywords:** Soft Drinks, Taxation, United Kingdom, Public Policy, Marketing, Public Health

## Abstract

The paper by Forde et al provides a useful qualitative consideration of marketing responses to the implementation of the 2018 Soft Drinks Industry Levy (SDIL) in the United Kingdom. This commentary discusses that paper and its conclusions and seeks to place them in a broader context for marketing, fiscal measures and health and public policy. It suggests that modern conceptualisations of marketing and wider considerations of market and non-market strategies could provide a valuable lens to understand the ways in which companies and sectors respond to the threats they perceive and the constantly changing sectoral opportunities. It is important that fiscal measures introduced have the desired effects, and that not only positive behaviours (whether of companies or consumers) are incentivised, but that adverse behaviours are actively disincentivised.

## Introduction

 There has been rising concern over the contribution of commercial activities to a growing health crisis, including of obesity and diabetes. Manufacturers, retailers and other product suppliers have been identified as selling, and promoting increased consumption of, products which can be damaging to personal health. Products such as cigarettes and alcohol are well known long-standing examples and increased taxation on them has been one component of the policy response. More recently, concerns over products high in saturated fats, sugar and salt have begun to attract more attention, given their direct link with individual and population health.

 In a number of countries this has led to the development of fiscal measures including taxation on soft drinks and in particular sugar-sweetened beverages. Such measures are identified as one lever — and possibly the most appropriate —to affect behaviours, both of firms and of consumers. In the United Kingdom this aim of changing the market led to the introduction of the Soft Drinks Industry levy (SDIL) in April 2018.

## Understanding Marketing Responses to SDIL

 The recent paper in this journal^1^ attempting to understand marketing responses to the SDIL is the focus of this commentary. That paper sought to understand whether tax design and public policy could be enhanced through a better understanding of the corporate reactions to SDIL. Qualitative interviews with eighteen academic, industry and civic society stakeholders focused on the marketing responses to the SDIL and the development of a theoretical framework. This framework suggests the marketing response, accelerated rather than precipitated by the SDIL, was co-ordinated and context specific, raising issues about potential predictability and thus more targeted or nuanced policy-making.

## Limitations With the Study

 The more academic work that is undertaken in this broad area, the better, especially when it is conceptually and theoretically grounded and undertaken rigorously. This is the case with this paper, and it adds to our growing understanding of the corporate, sector and market responses to interventions affecting commercial operations. There are though some limitations with this study (as with any). Three can be noted:

 First, there is an issue over the primary research and the interviews/interviewees that form the core of this paper. The initial ambitions around the scope and depth of interviewees proved unattainable and despite statements around primary research interview saturation, there are concerns over the mix and depth of the interviews. This is recognised by the authors, but the results need considering in terms of this limitation. The lack of a range of “insider” respondents is a gap. Alternative longer-term approaches may have produced a deeper and broader view of marketing responses.

 Secondly, the conceptualisation of marketing is in terms of the 4Ps model (product, price, place, and promotion). This is a rather dated and limited view of the range and role of marketing, and a broader view might have produced an enhanced consideration of how companies responded. More modern conceptualisations of marketing have been developed^2^ as for example in service-dominant logic^3^ and customer dominant logic.^4,5^ Their use might have generated a more extensive consideration of the marketing responses to SDIL. Businesses and consumers are bound-up in the product characteristics, use and meaning and customers are not neutral recipients of corporate decisions. This is obvious in the brand-heavy soft drinks market, where marketing goes well beyond the 4Ps.

 This wider approach could also have discussed the ways companies co-opted consumer concerns to limit the scope of the SDIL before its introduction, how consumer dissent played out after introduction, and how companies reacted to this. More widely, considerations of corporate market and non-market strategies^6-8^ including organised opposition to, and engagement with, the introduction of the SDIL over a sustained period would have shown how soft drink companies (and others) try to shape the sector to their advantage. The responses to implementation might have been more meaningfully interpreted as part of a continuum of market and non-market strategies in this “battle.” Profit does not depend on the 4Ps alone, but on the shape and structure of the market in widest sense.

 Thirdly, these concerns over conceptualisation and primary research suggest that a slightly functional view of the market is being expressed in the paper. By focusing on the 4Ps the emphasis is placed on an activity or a change in characteristics. This leads to a stress on a point rather than a process and perpetuates a narrower view of what companies and their marketing teams do, than is the reality. The concern is focused on the reaction and its “acceleration” rather than seeing this as part of the longer-term interactions of market management.

 These comments are not meant to negate the contribution or findings of the paper but to suggest that a broader and deeper approach to conceptualisation and primary research may build on this base and add further value and understanding. Specific case studies, probably longitudinal, might help in this.

 As an illustration of this, it is notable that the role of the retailer in the corporate reactions to SDIL was relatively under-explored. Retailers’ relationships with suppliers can be contentious as recent public disagreements in the United Kingdom between Tesco and Kraft Heinz have shown and the relative power dimensions are significant. As noted by one of the respondents in the paper, there are issues arising not only over price but also in-store positioning. In this context though most leading retailers also use their own brands (private label/retailer brands) to frame the category and specifically the manufacturer brand products, both in price and positioning. Retailers had to factor SDIL into their direct thinking and responses to their operations and in dealings with suppliers. Retailers though are also not the only supply channel for consumers in soft drinks and consideration of differential responses in retail and other purchase situations could have been of interest eg, hospitality and home delivery, though margins here may make the issue less relevant in some situations. The focus on the manufacturer responses in terms of the 4Ps means that the shifts within and amongst purchase points for consumers is perhaps underplayed.

## The Broader Context

 There is also learning beyond the soft drinks sector. As noted earlier, alcohol and cigarettes are well known, and long-standing examples of taxation being used to increase price to reduce consumption. Both product categories, to varying degrees, have also seen restrictions on packaging and promotion and attempts to break the lifestyle/co-creation linkages. Within this, the introduction of minimum unit pricing (MUP) on alcohol in Scotland possibly demonstrates some different dimensions to policy and its impacts that could potentially “read across” to sugar-sweetened beverages. The extensive research programme into the introduction and effects of MUP on consumer and business behaviour in Scotland, and the ability to compare Scotland with England (where the policy does not apply) is providing valuable information. A steady stream of research publications^9^ has become available and the final evaluation will be published in 2023/2024 ahead of Scottish Parliamentary scrutiny of policy maintenance, change or abolition.

 In this MUP context it is also worth noting the extensive attempts to stop the fiscal measure, including a long running legal battle prior to implementation which delayed MUP for approximately six years. The industry warned of adverse consequences on introduction for businesses and on consumer behaviours and health. The evidence thus far has shown this to be unfounded and indeed some unexpected benefits in competition terms have been identified. This is an affirmation of the comment in the paper that “the idea that this was some kind of financial catastrophe has proved very untrue” (p. 5). This is significant as it illustrates the point about process and the non-market strategies that companies (or indeed sectors) engage with. Attempts are made to stop, divert/delay or weaken policy, often with dire warnings about commercial implications; these are later mostly seen to be unfounded. The responses identified in the paper are a latter part of this continuum of resistance and change and need to be reviewed in this light.

 The example also serves as a reminder that the market is not static, neither from a business nor a consumer perspective. This is embedded in the paper, but one aspect identified perhaps deserves further consideration. One “reaction” to the SDIL was the alteration of product portfolios by companies. Some of this was achieved internally through reformulation and new product development (NPD). The NPD process is a continual one as companies scan the market, identify and react to (or in some cases lead) trends in needs and wants. How much NPD was triggered by SDIL (prior to and after implementation) and how much would have occurred through natural responses to this market scanning of consumer trends and concerns, remains unclear. Personal health was an emerging trend and so change would perhaps have been expected anyhow, though probably polarised in socio-demographic terms. There is also though an external way of altering portfolios, as noted in the paper. Larger companies bought up smaller emerging businesses to add a non-sugar–sweetened offer to their own portfolio. Again, this could be a natural response to trends and sales analyses. It does show however the ever-present restructuring of the market that needs closer attention. Whether start-ups which are successful can continue to be scaled-up in a larger organisation is an open question or whether the point of the “capture” can be to circumscribe the growth and thus manage the overall market.

 There is also a global aspect to consider. Many organisations operate across the globe, including in developing countries. The context in such countries may be different from developed countries, as are perhaps the health priorities. There may be a question over applicability in different circumstances over measures such as SDIL. The example of cigarettes also suggests that as restrictions rise in developed countries, so marketing and focus turns to developing countries. Lessons may be transferred by both corporate actors and by public health bodies.

## Concluding Remarks

 The rising concern over commercial impacts on personal and population health have led to interventions in market and company operations. Resistance to such interventions are part of the strategic corporate response and actions need to be considered in that light. The evidence shows that companies cannot be relied upon to consider such health issues fully. As such it is incumbent to understand what measures work and what different impacts they have in meeting population health goals. Measures need to be targeted and focused both to attain their goals, but also need to be designed to incentivise positive behaviours (consumer or corporate) and dis-incentivise adverse behaviours. The market is a social construction and is not neutral nor a level playing field. Health policy needs to be designed with this fully in mind and with combinations of measures considered and applied. Learning from SDIL, via this paper and the wider evaluation, is an important step in developing this understanding.

## Ethical issues

 Not applicable.

## Competing interests

 I am a member of the Economic Advisory Group for the Economic Evaluation of Minimum Unit Pricing in Scotland, which is referenced in the commentary. This is an unpaid role.

## References

[1] Forde H, Penney TL, White M, Levy L, Greaves F, Adams J (2022). Understanding marketing responses to a tax on sugary drinks: a qualitative interview study in the United Kingdom, 2019. Int J Health Policy Manag.

[2] Vargo SL, Lusch RF (2004). Evolving to a new dominant logic for marketing. J Mark.

[3] Tregua M, Brozovic D, D’Auria A (2021). 15 years of service-dominant logic: analyzing citation practices of Vargo and Lusch (2004). J Serv Theory Pract.

[4] Heinonen K, Strandvik T (2015). Customer-dominant logic: foundations and implications. J Serv Mark.

[5] Anker TB, Sparks L, Moutinho L, Grönroos C (2015). Consumer dominant value creation: a theoretical response to the recent call for a consumer dominant logic for marketing. Eur J Mark.

[6] Wood B, Williams O, Nagarajan V, Sacks G (2021). Market strategies used by processed food manufacturers to increase and consolidate their power: a systematic review and document analysis. Global Health.

[7] Eastmure E, Cummins S, Sparks L (2020). Non-market strategy as a framework for exploring commercial involvement in health policy: a primer. Soc Sci Med.

[8] Lacy-Nichols J, Williams O (2021). “Part of the solution”: food corporation strategies for regulatory capture and legitimacy. Int J Health Policy Manag.

[9] Overview of Evaluation of MUP. Public Health Scotland. http://www.healthscotland.scot/health-topics/alcohol/evaluation-of-minimum-unit-pricing-mup/overview-of-evaluation-of-mup/why-we-are-evaluating-mup. Updated April 6, 2022. Accessed May 26, 2023.

